# Cold comfort: Arctic seabirds find refugia from climate change and potential competition in marginal ice zones and fjords

**DOI:** 10.1007/s13280-021-01650-7

**Published:** 2021-11-09

**Authors:** Anne-Sophie Bonnet-Lebrun, Thomas Larsen, Thorkell Lindberg Thórarinsson, Yann Kolbeinsson, Morten Frederiksen, Tim I. Morley, Derren Fox, Aude Boutet, Fabrice le Bouard, Tanguy Deville, Erpur Snær Hansen, Thomas Hansen, Patrick Roberts, Norman Ratcliffe

**Affiliations:** 1 British Antarctic Survey (BAS), High Cross, Madingley Road, Cambridge, CB3 0ET UK; 2 Department of Archaeology, Max Planck Institute for the Science of Human History, Kahlaishce Str. 10, 07745 Jena, Germany; 3 Icelandic Institute of Natural History, Garðabær, Iceland; 4 Northeast Iceland Nature Research Centre, Húsavík, Iceland; 5 Department of Bioscience, Aarhus University, Aarhus C, Denmark; 6 South Iceland Nature Research Centre, Ægisgata 2, 900 Vestmannaeyjar, Iceland; 7 GEOMAR Helmholtz-Zentrum Für Ozeanforschung, ZLCA, Düsternbrooker Weg 20, 24105 Kiel, Germany

**Keywords:** Climate change, Competition, Fjords, Niche partitioning, Refugia, Sea ice

## Abstract

Climate change alters species distributions by shifting their fundamental niche in space through time. Such effects may be exacerbated by increased inter-specific competition if climate alters species dominance where competitor ranges overlap. This study used census data, telemetry and stable isotopes to examine the population and foraging ecology of a pair of Arctic and temperate congeners across an extensive zone of sympatry in Iceland, where sea temperatures varied substantially. The abundance of Arctic Brünnich’s guillemot *Uria lomvia* declined with sea temperature. Accessibility of refugia in cold water currents or fjords helped support higher numbers and reduce rates of population decline. Competition with temperate Common guillemots *Uria aalge* did not affect abundance, but similarities in foraging ecology were sufficient to cause competition when resources are limiting. Continued warming is likely to lead to further declines of Brünnich’s guillemot, with implications for conservation status and ecosystem services.

**Supplementary Information:**

The online version contains supplementary material available at 10.1007/s13280-021-01650-7.

## Introduction

The persistence of species through space and time is constrained by a suite of environmental conditions that support survival and reproduction, defined as the fundamental niche (Pulliam 2000). Intra-specific competition imposes additional constraints, producing the realised niche, in which geographic projection comprises a species’ observed distribution (Pulliam 2000). Climate change studies mostly address how niches shift in space through time for single species in isolation but are increasingly finding that inter-specific competition can influence the shape and magnitude of climate responses (Helland et al. 2011; Milazzo et al. 2013; Stenseth et al. 2015).

The Arctic Ocean has experienced increased inflow of warm Atlantic water, which has contributed to the melting of sea ice and altered zooplankton and fish community composition (Post et al. 2013; Fossheim et al. 2015). This has in turn led to impacts upon the population dynamics of higher vertebrate predators in the Arctic, including seabirds (e.g. Descamps et al. 2013; Hovinen et al. 2014). Brünnich’s guillemot *Uria lomvia* and Common guillemots *Uria aalge* (hereafter BG and CG) are sister species with similar life histories and niches (Barrett et al. 1997). They are broadly segregated by temperature; BG is a high-latitude species that breeds on cliffs adjacent to waters between − 2 and 8 °C while CG replaces them at lower latitudes where waters are between 2 and 16 °C (Irons et al. 2008). BG is adapted to exploit sympagic prey species in cold, ice-covered habitats while CG prefers to feed on schooling fish in warmer, open water (Irons et al. 2008). They breed sympatrically at colonies in the low- and sub-Arctic, where they have become a classic case study of inter-specific competition (Barrett et al. 1997). CG tends to dominate over BG for nest sites and food within areas of sympatry (Williams 1974; Barger and Kitaysky 2012), which raises the possibility that competition from CG may exacerbate climate impacts upon BG at the trailing edge of its range.

In this study, we examine the population and foraging ecology of BG and CG in Iceland, which represents their largest zone of sympatry in the Atlantic (Irons et al. 2008). Iceland represents an ideal study area as ocean currents of differing provenance converge there to create levels of habitat variability that typically occurs across ocean basins (Astthorsson et al. 2007). A regime shift in the mid-1990s due to inflow of warm Atlantic water has altered food webs (Valdimarsson et al. 2012), with associated population declines of 30% for CG and 44% for BG between the early-1980s and mid-2000s (Garðarsson et al. 2019). We explore the impact of spatiotemporal environmental variability upon the abundance and foraging ecology of the two guillemot species, and assess evidence for inter-specific competition. Specifically, we tested the hypotheses that (i) BG numbers and relative abundance would decrease with SST and competitive pressure from CG, using census data, and (ii) that BG would attempt to reduce competition with CG by segregating into different habitats, using data from biologging and stable isotope ratios in blood samples.

## Methods

### Study sites

**Fig. 1:**
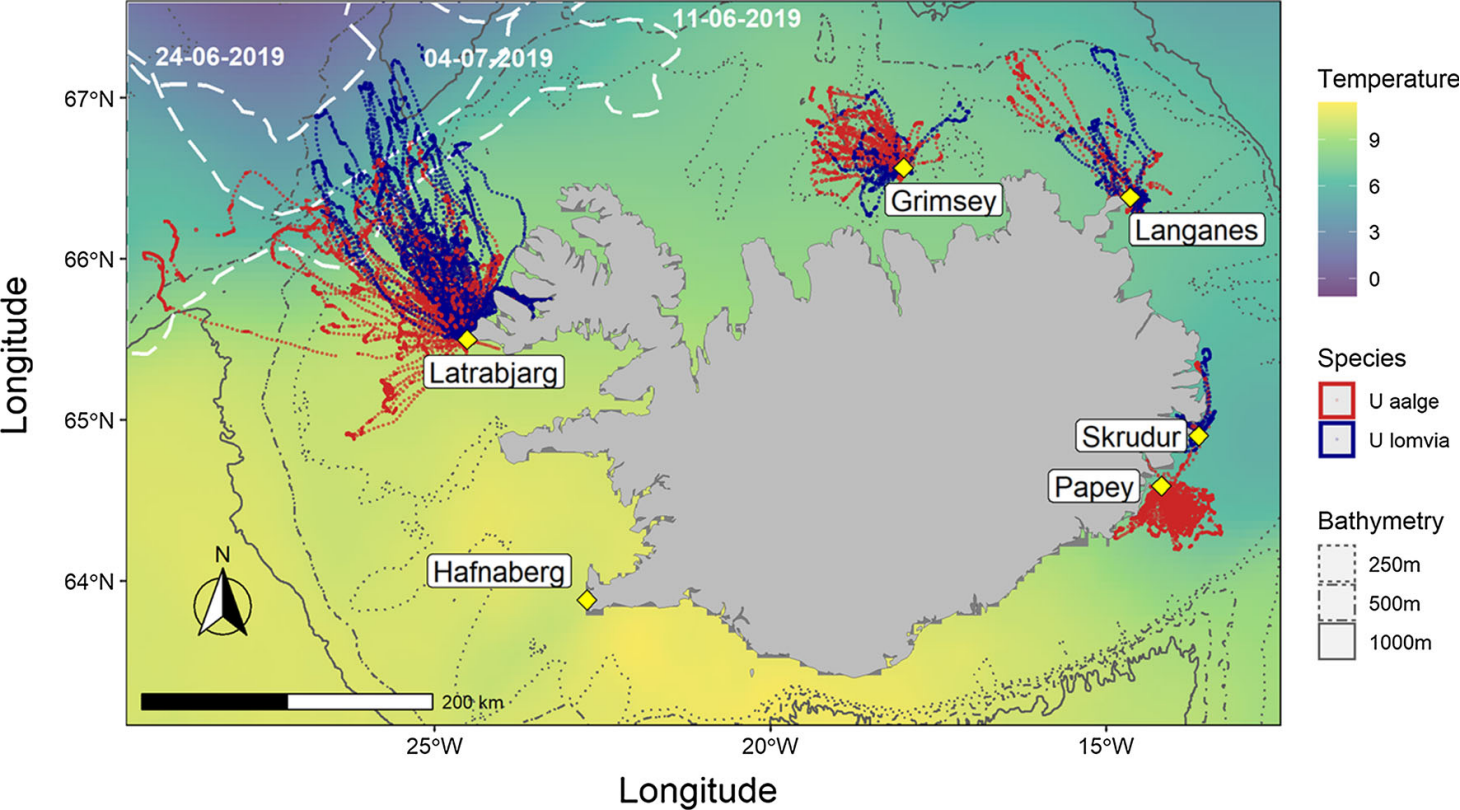
Map of the raw tracking data by species plotted on an Equal-Area Scalable Earth Grid projection (ESPG: 3408). Large dots of tracks indicate foraging segments and small ones the path interpolated at a constant 3 min interval. Yellow diamonds are the colony locations. White-dashed lines indicate the position of the ice edge (external limit of the Marginal Ice Zone) at the start, the middle and the end of the study. Background colours correspond to sea surface temperature averaged across the study period in 2019

Data were sampled from seven guillemot colonies around Iceland (Fig. 1). Látrabjarg (65.50° N, 24.52° W) in the NW is surrounded by the warm Irminger Current but is also within commuting range of the cold East Greenland Current. Grímsey (66.57° N, 18.02° W) is an island north of Iceland in the Irminger Current, but also within range of the cool East Icelandic Current. Langanes (66.38° N, 14.54° W) is a peninsula at the northeast tip of Iceland at the front between the Irminger Current and East Icelandic Current. Skrúður (64.90° N, 13.62° W) and Papey (64.59° N, 14.18° W) are two islands 43 km apart off the SE of Iceland situated in the cool East Icelandic Current. Papey is ~ 35 km NE of the front between the East Icelandic Current and the warm North Atlantic Current. Hafnaberg is a small mainland cliff in the SW of Iceland (63.75° N, 22.75° W) in the North Atlantic Current (see Astthorsson et al. 2007 for details of oceanography). The last published census (from 2006 to 2008; Garðarsson et al. 2019) estimated populations of 344,000 pairs of both species combined at Látrabjarg (34.3% BG), 71,400 at Grímsey (5.7%), 46,800 at Langanes (6.2%), 13,000 at Skrúður (11.5%), 3700 at Papey and 419 at Hafnaberg (which hosted ~ 5 and no BG pairs in 2019, respectively).

### Data collection

We tracked the movements of BG and CG from their colonies at Látrabjarg, Grímsey, Langanes and Skrúður in 2019. Only CG were available to sample on Papey. Breeding birds were captured using a noose pole and equipped with a nanoFix GPS logger (Pathtrack, Otley, UK, 6.1 g, < 1% of the average bird mass) and G5 Time Depth Recorder (TDR: CTL, Lowestoft UK, 2.7 g). GPS devices were attached to the feathers over the synsacrum and TDRs to those over the keel, using waterproof tape and glue. Larger GPS devices (18 g) had no effect on the time budgets or diving performance of CG in Sweden (Evans et al. 2020). GPS devices recorded locations every three minutes and TDRs temperature every second. Birds were recaptured to retrieve the devices several days later. A total of 113 CG and 60 BG were equipped between 11 June and 4 July 2019 (late incubation and chick rearing; see Fig. S1 for deployment details). We recovered a total of 88 GPS and 92 TDR from CG and 48 GPS and 50 TDR from BG. Losses were due to birds evading recapture and plucking off loggers. The tracks at Langanes were augmented by GPS data collected in 2015 (using GT-120 GPS loggers; Mobile Action, Taipei, Taiwan; 18 g) and 2020.

We sampled one ml of blood by venepuncture for stable isotope analysis. Plasma and cells were separated in a Micro-Star 12 centrifuge (VWR, Leuven, Belgium) at 12,300×*g* for 4 min, then placed in glass petri dishes, and dried in a desiccator for four to six days. Samples were collected during 2018 and 2019 at all sites except Hafnaberg (2018 only; in 2019, birds were hidden beneath an overhang) and Skrúður (2019 only; access was not granted in 2018). Blood was sampled randomly in 2018 and from tracked birds upon recapture in 2019.

### Stable isotope analysis

The ratios of nitrogen stable isotopes provide information on the trophic level at which an animal feeds and that of carbon the habitats in which it forages (inshore vs. offshore), although caution is required with interperation as baseline ratios may vary through space and time (Thompson et al. 1999; Hansen et al. 2012). Isotopes in blood cells describe diet and habitat use in the month prior to collection (Hobson and Clark 1993). Isotope data are expressed in delta (*δ*) notation:

$$ \delta^{i} E_{{{\text{sample}}}} = \frac{{\left( {\frac{{i_{E} }}{{j_{E} }}} \right)_{{{\text{sample}}}} - \left( {\frac{{i_{E} }}{{j_{E} }}} \right)_{{{\text{ref}}}} }}{{\left( {\frac{{i_{E} }}{{j_{E} }}} \right)_{{{\text{Ref}}}} }}. $$

For the element *E*, the ratio of heavy (i) to light (j) isotope is measured in both sample and references. To express the isotopic data as per mil (‰), they are multiplied by 1000. The isotope ratios are expressed relative to international standards; Vienna Pee Dee Belemnite (VPDB) for carbon and atmospheric air for nitrogen.

Elemental content and bulk isotope values of blood cells were determined at the Stable Isotope Facility of the Experimental Ecology Group, GEOMAR, Kiel. Approximately 60–100 µg blood cell dry mass of each sample was weighed into small tin capsules (3.2 × 4.0 mm). Samples were analysed by a customised, high sensitivity elemental analyser connected to a stable isotope ratio mass spectrometer (DeltaPlus Advantage, Thermo Fisher Scientific, Germany) as described by Hansen and Sommer (2007). System calibration was implemented by the combustion of International Atomic Energy Agency (IAEA-N1, IAEA-N2, IAEA-N3 for *δ*^15^N) and National Institute of Standards and Technology (NBS-22 and NBS-600 for *δ*^13^C) compounds. Acetanilide p.a. (Merck, Germany) was used as an internal standard after every sixth sample within each sample run. The overall standard deviation (SD) for the low measurement range 2.5–8.0 µg N and 5.0–80 µg C was ± 0.25‰ and ± 0.2‰, respectively. The overall SD for the higher measurement range 5.0–15.0 µg N and 10.0–140 µg C was ± 0.2‰ and ± 0.15‰, respectively.

### Data analysis

Full details of analytical methods are provided in Supplement S1. The log-transformed abundance of each guillemot species at a given time and location (*n*_*ti*_) across all colonies in Iceland in 1983–1986 and 2005–2008 was taken from Garðarsson (1995) and Garðarsson et al. (2019). These were modelled in relation to cliff area (*A*_*i*_; from Garðarsson 1995), competitor abundance (*C*_*i*_), and the average or minimum sea surface temperature within the foraging area of each colony during each survey period (see Supplement S1 and Fig. S2 for details of SST derivation). A random intercept for site (*u0*_*i*_) was fitted to accommodate repeated measures. Within subject centring (Curran and Bauer 2011) was used to disaggregate the time and space components of SST effects on abundance. The mean SST within a colony (*z̄*_*i*_) represents between-site variation, and the deviations of SST at each time point from the site mean (*ż*_*ti*_) represents within-site variation. The global model was

$$ n_{ti} = \left( {\gamma 00 + \gamma 01\overline{z}_{i} + \gamma 10\dot{z}_{ti} + A_{i} + C_{i} } \right) + \left( {u0_{i} + r_{ti} } \right). $$

The linear mixed models (LMM) were fitted using an identity link and normal errors. The proportion of BG within colonies was modelled in relation to minimum SST using a generalised LMM with a logit link and binomial errors to examine changes in relative abundance of the two species with SST.

GPS tracks were split into foraging trips, and the maximum distance travelled from the colony during each was calculated. Foraging segments of trips were classed as periods when travel speed was slow, temperature was uniformly low, and diving activity occurred, based on visual inspection of GPS and TDR data (Tremblay et al. 2003). The locations at which foraging segments began were estimated from linear interpolation, based on the time the bird alighted on the water (taken as a sudden decrease in TDR temperature; Tremblay et al. 2003) relative to the times of the previous and subsequent GPS fixes. Surface temperatures from the TDRs were averaged within each foraging segment.

We classed sites according to the sector of Iceland in which they are situated (SW, NW, N, NE and SE). Papey and Skrúður in the SE were, thus, combined for further analysis. We modelled the response variables (trip distance, SST in foraging segments and isotope ratios) using general least squares or mixed models with an identity link and normal errors. Explanatory fixed factors were species, colony and (for isotopes only) year, while random effects were individual (for trip distance and SST in foraging segments only). As there were missing site/species/year combinations for the stable isotope sampling, each of the site-species-year combinations were expressed as levels of a single factor. As heteroscedasticity was evident among factor levels for all responses, we fitted identity variance structures to meet model assumptions and estimate differences in the variability among groups (the number of standard deviations relative to a reference level; SD_r_). In the case of SST in foraging segments, serial autocorrelation was evident in the residuals, so an order-one auto-regressive term was fitted within individual.

In all analyses, Akaike's Information Criterion, adjusted for small sample size (AIC_c_) was used for model selection and *R*^2^ were used to assess model fit. For models with random effects, both marginal $R_{m}^{2}$ (fixed effects alone) and the conditional $R_{c}^{2}$ (fixed and random effects combined) were calculated. Tukey HSD was used to test differences between factor levels of interest (species, year and sites while controlling for each of the others).

## Results

### Patterns of abundance in relation to SST

**Fig. 2:**
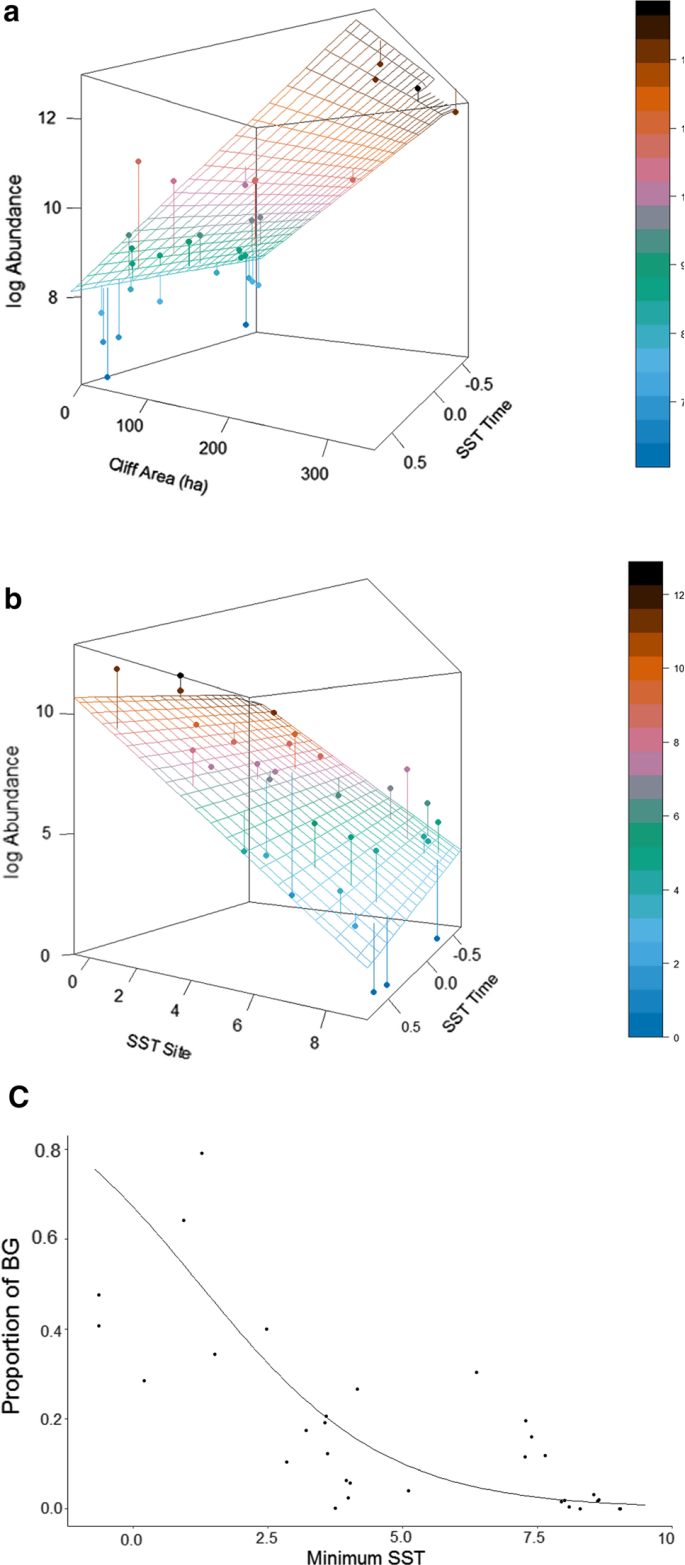
Abundance of **a** Common Guillemot (CG) in relation to change in average SST (°C) within sites (SST time) and cliff area; **b** Brünnich’s guillemot (BG) in relation to minimum SST variation between colonies (SST site) and its change within sites and **c** the proportion of BG in colonies in relation to minimum SST. Points are raw data and the lines are fitted values from mixed models

For CG abundance, models with an effect of cliff area received support, but those with an effect of BG competitor numbers did not (Table S1). Models including the average SST received more support than those with the minimum SST, which was due to an effect within sites through time as that between sites was marginal (Table S1). Log-transformed CG numbers showed a negative response to average within-site SST (slope = − 0.456, SE = 0.096) and a positive one with standardised cliff area (slope = 1.47, SE = 0.288), such that spatial variation in abundance was explained by availability of cliff breeding habitat and trends by changing SST (Fig. 2a). The $R_{m}^{2}$ for this model was 0.649 and $R_{c}^{2}$ 0.985.

For BG abundance, models with minimum SST within the foraging area received more support from AIC_c_ than those with average SST, while those including effects of cliff area or CG competitor numbers were not supported (Table S2). The minimum SST effect was evident between and within sites, showing it affected both colony size and trends (Table S2). The selected model revealed that numbers of BG showed a negative response to SST that was steeper within sites (slope = − 1.687, SE = 0.488) than among them (slope = − 0.948, SE = 0.157). However, the range of SST was far greater among sites than within them, such that spatial variation in SST had the greatest overall influence on abundance (Fig. 2b). The $R_{m}^{2}$ of this model was 0.679 and the $R_{c}^{2}$ 0.903.

The proportion of BG at a colony showed a negative response to the minimum SST within the foraging range (ΔAIC SST vs. Null model = 12.07, SST model AICc weight = 0.998; Fig. 2c). The $R_{m}^{2}$ of the SST model was 0.50 and the $R_{c}^{2}$ 0.999.

### Foraging distribution, range and habitat use

Maps of the spatial distribution of foraging trips from the study colonies are shown in Fig. 1 and Fig. S3. CG at Látrabjarg foraged across the Irminger Current, while BG oriented trips toward the NNW and the East Greenland Current. Of the foraging segments for BG at Latrabjarg, 27% (*N* = 373) were in waters below 5.5 °C that indicated use of the East Greenland Current, compared to 9.1% (*N* = 339) for CG. Relatively high use of the Arnarfjörður fjord was also evident for BG at Látrabjarg (BG 19.9% and CG 7.3% of foraging segments). Birds at Grímsey oriented trips to the deeper water (> 250 m deep) to the NW of the island. Birds at Langanes mostly performed short foraging trips in 2019 and 2020 but also made some longer ones in 2015. Birds from Skrúður performed nearshore foraging trips and showed particularly high use of the Reyðarfjörður fjord (BG 42.1% and the single CG 100%). CG at Papey mostly foraged offshore of the colony and only 1.2% of foraging was in fjords.

Both the mean and variance of maximum foraging range differed by site but not species (Table S3). The $R_{m}^{2}$ of this model was 0.297 and the $R_{c}^{2}$ 0.560. Foraging trips in 2019 were the shortest in the NE (11.5 km, SE = 0.14, SD_*r*_ = 1), longer but less variable in the SE (17.7, 0.06, 0.69) and the N (27.1, 0.12, 0.89) and longest and most variable in the NW (50.2, 0.09, 1.73).

The mean and variability of SST in foraging segments was affected by an interaction between species and region (Table S3). The $R_{m}^{2}$ of this model was 0.388 and the $R_{c}^{2}$ 0.487. The estimates show that waters used by CG were coolest in the SE, intermediate in the N and NE, and warmest but most variable in the NW (Table 1). When compared to CG at the same sites, waters used by BG were cooler and more variable in the NW but warmer in the SE (Table 1).

**Table 1:** SST utilisation by guillemots in Iceland in relation to species and sector of the coast where the colony was situated, sampled using bird-borne temperature loggers. SST is the mean SST used with one standard error. SD_*r*_ indicates the number of standard deviations scaled relative to CG in the NE, which is an estimate of relative variability among species and sites. The *z* score and *P* value show the significance of Tukey HSD pairwise tests between species within sites. *N* is the number of foraging segments at which SST was sampled

Sector	Species	N	Mean	SE	SD_*r*_	*z*	*P*
NW	CG	339	8.49	0.163	2.36	2.64	< 0.01
NW	BG	373	7.82	0.192	3.55		
N	CG	218	7.26	0.178	0.55	1.54	> 0.1
N	BG	68	7.77	0.277	0.46		
NE	CG	10	8.00	0.574	1.00	0.44	> 0.6
NE	BG	11	7.68	0.457	0.85		
SE	CG	388	4.85	0.130	0.64	2.89	< 0.005
SE	BG	61	5.74	0.281	0.66		

### Stable isotope ratios

**Fig. 3:**
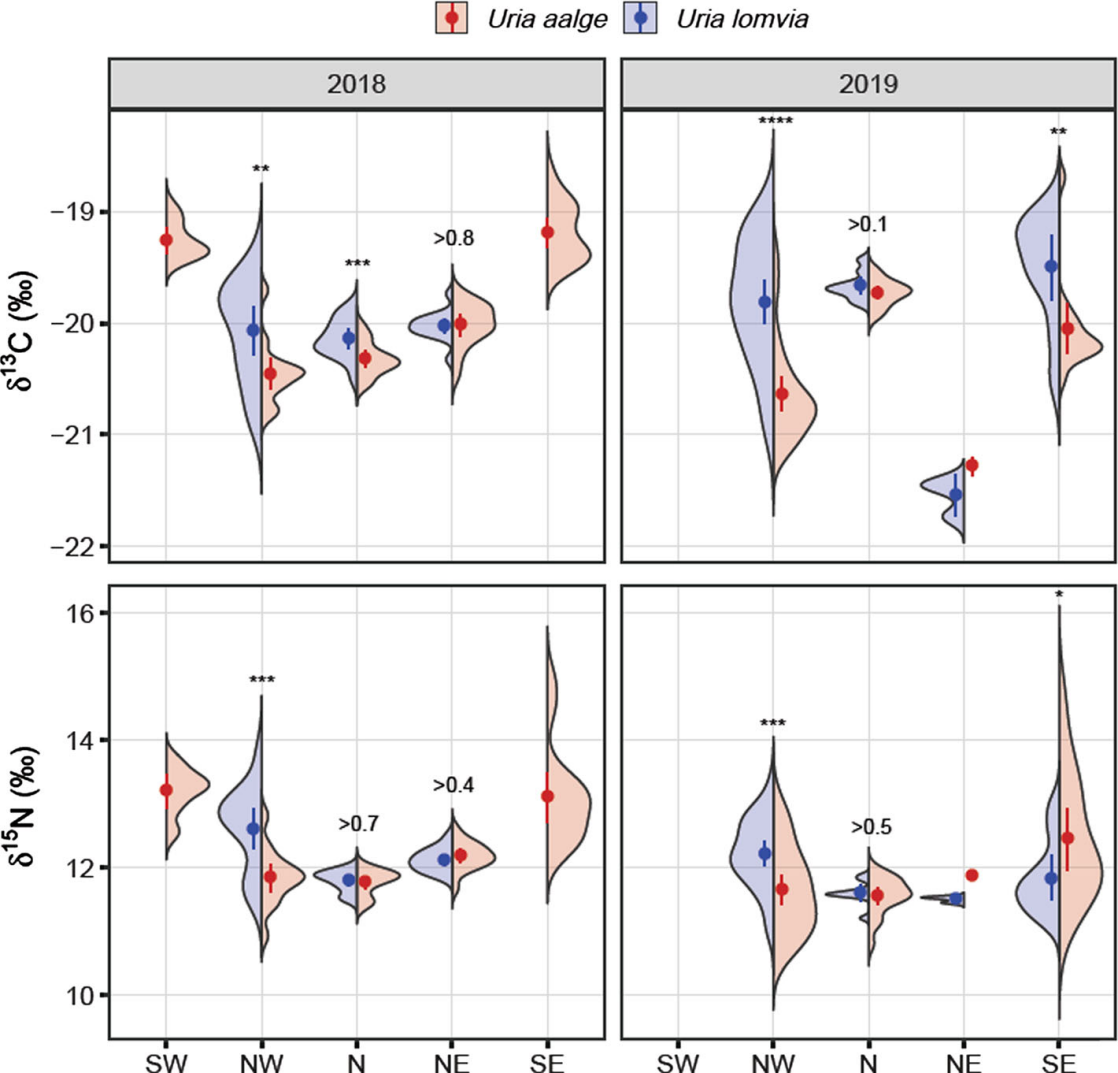
Split violin plots of δ^13^C and δ^15^N in guillemot blood cells. Violins represent the kernel density of the frequency distribution of the given group and isotope. Points are means with one standard error. Asterisks denote significance of species differences (* < 0.05, ** < 0.01, **** < 0.0001)

The means and variances of the species-site-year groupings differed for both δ^13^C and δ^15^N (Table S3). The *R*^2^ of these models was 0.538 and 0.478, respectively. For CG in 2018, both δ^13^C and δ^15^N ratios were significantly higher in the SW and SE compared to the northern sectors (Fig. 3; Tables S4 and S5). This regional pattern was not evident in 2019 as samples from the SW were unavailable and δ^13^C for the eastern sectors declined while those in the N and NW increased (Table S6, Fig. S4), which was associated with a cooling of the East Icelandic Current and warming of the Irminger Current in 2019 (Fig. S5). Variability of δ^13^C and δ^15^N was higher for both species in the NW and SE (Fig. 3, Table S4).

Comparisons of species within sites and years showed that BG in the NW had higher δ^13^C and δ^15^N values compared to CG in 2018 and 2019 (Fig. 3, Table S4). The BG frequency distributions showed strong skews towards low δ^13^C and δ^15^N compared to CG, which indicate different diets or habitat use by the two species (Fig. 3). In the N, BG had higher δ^13^C than CG in 2018 but not 2019, while δ^15^N was similar in both years (Fig. 3, Table S4). In the SE, BG had significantly higher δ^13^C and δ^15^N in 2019, but this is confounded with site as BG were sampled only at Skrúður and CG mostly at Papey.

## Discussion

This study presents multiple, complementary lines of evidence that elucidate the relative importance of climate change and potential competition for congeneric Arctic and temperate seabird species across an extensive zone of sympatry. Census data revealed the influence of SST on distribution and trends, while the tracking and isotope data revealed how the observed patterns could be explained by habitat use and behaviour. Stable isotopes not only acted as markers for different water masses which could arise from their different baselines or prey species but were also affected by localised habitat use (Thompson et al. 1999; Hansen et al. 2012). The tracking data provide detailed information on habitat use that allowed the sources of variability in isotope ratios to be identified. On the other hand, tracking data were of relatively short duration, so the isotope sampling allowed confirmation of the consistency of habitat use during the month prior to sampling and across years.

The abundance of the Arctic BG showed a strong negative relationship with the minimum SST within their foraging areas through both time and space, which explains why they have always been relatively rare in the south of Iceland and why numbers declined following the regime shift in the mid-1990s (Garðarsson et al. 2019). In the North Atlantic, water masses from lower latitudes are more δ^13^C enriched than those from higher latitudes (Thompson et al. 1999; Hansen et al. 2012), so it is telling that CG blood cells sampled from colonies in the SE and SW in 2018, where BG have virtually disappeared, showed much higher δ^13^C than northern colonies where BG have persisted. The decline in BG numbers across Iceland and particularly in the south (Garðarsson et al. 2019) is, therefore, consistent with a contraction in the geographic projection of the species’ fundamental niche in response to warming. Irons et al. (2008) also found that sudden changes in temperature were associated with guillemot population declines at a global scale.

BG absolute and relative abundance was highest and most stable through time in the NW of Iceland (Garðarsson et al. 2019), even though the average SSTs here were relatively high. However, the minimum SSTs were the lowest in Iceland owing to the accessibility of the cold water in the East Greenland Current. Our tracking data, alongside those from 1995 (Benvenuti et al. 1998), confirmed that BG from Latrabjarg commuted long distances to reach cold Arctic water and marginal ice zones in the East Greenland Current. The enriched δ^13^C of BG blood cells, compared to CG, in the NW are indicative of birds foraging in the marginal ice zone (Cusset et al. 2019) during the month prior to tracking, which would span the incubation and the pre-breeding seasons. The accessibility of cold water and ice margins has also been found to be positively related to geographic patterns of BG abundance in west Greenland (Laidre et al. 2008).

BG at Látrabjarg made intensive use of the Arnarfjörður fjord, and these habitats are known to be utilised as a foraging habitat by BG elsewhere (Mehlum et al. 2001). The accessibility of cold water in the northern fjords in the past is likely to explain the remarkably high proportion of BG in the colony at Drangey, as the East Greenland Current is unlikely to be accessible from this location (Fig. S2). BG and the single CG tracked from Skrúður used nearby Reyðarfjörður intensively and this, combined with the East Icelandic Current producing the lowest average SSTs of any guillemot foraging area in Iceland (Fig. S2), may have contributed to the rate of BG population decline there being among the slowest in the country (Garðarsson et al. 2019). In contrast, BG has almost disappeared from neighbouring Papey, where birds forage offshore in waters warmed by their proximity to the North Atlantic Current (Fig. S2).

As BG is an Arctic species, the cold water of the East Greenland Current and East Icelandic Current or fjords may act as refugia that allow populations to persist in a region that would otherwise be unfavourably warm. Cold refugia have been demonstrated to provide sanctuary for a wide range of high-latitude taxa as the climate warms (Hein et al. 2012; Morelli et al. 2017; Assis et al. 2018). However, the buffering effects of refugia may be overwhelmed by ongoing warming (Assis et al. 2018), and the long-term reduction in Arctic sea ice extent (Meier et al. 2014) along with the dramatic warming of the northern fjords in 2019 (Fig. S5) are of particular concern for BG population viability in the NW of Iceland.

We found no evidence for an effect of CG abundance upon that of BG. CG distribution was explained by availability of breeding habitat, but their numbers declined within site in response to warming SST as did BG, albeit at a slower rate. This is the opposite pattern to that expected if warming were causing CG numbers to grow and squeeze the realised niche of BG. The finding that variability in environmental conditions or food availability is more important than competition in population regulation accords with a long-term study of BG and CG at colonies in Russia (Durant et al. 2012) and moths in the UK (Mutshinda et al. 2011). However, evidence for competition effects that interact with climate has been found in European songbirds (Stenseth et al. 2015) and fish (Helland et al. 2011; Milazzo et al. 2013).

The lack of a negative effect of CG upon BG populations could arise from niche partitioning that allows the two species to coexist (Hutchinson 1957). Telemetry data showed increasing foraging range of guillemots in relation to colony size which is likely to be due to competition for food (Gaston et al. 2013). The fact that BG had similar foraging ranges to CG, despite being far less numerous, suggests that the per-capita strength of inter-specific competition is similar to that of intra-specific competition. Within their shared foraging ranges, niche partitioning among the two species according to SST or isotope ratios was weak at all but one site, suggesting that birds used similar habitats and consumed similar diets. This creates the potential for high inter-specific competition when resources become limiting unless niche partitioning increases. Patterns of niche partitioning among the two species according to diet (Birkhead and Nettleship 1987; Barrett et al. 1997; Barger and Kitaysky 2012) or space use (Linnebjerg et al. 2013; Kokubun et al. 2016; Pratte et al. 2017) are very variable across studies, which suggests that the two species adapt their segregation tactics in response to local and ephemeral patterns of prey distribution and competitor behaviour.

Segregation between the two species in environmental space was found at Látrabjarg. Tracking and isotope data revealed that CG largely remained within the warm Irminger Current, while BG also utilised colder waters in the East Greenland Current. It is, therefore, possible that BG are selecting cold water and ice margins to avoid competition with CG, or they might have evolved to exploit colder water in isolation from CG, and prefer to use such habitats in regions of secondary contact where these are available. Both explanations can account for spatial segregation in natural systems; for example, competition explains segregation of sympatric nightingales *Luscinia* sp. in NE Europe (Reif et al. 2018) while habitat preference explains that of martens *Martes* sp in Poland (Wereszczuk and Zalewski 2015). However, BG at Látrabjarg also foraged extensively in the Irminger Current *en route* to the East Greenland Current (Fig. 1), which resulted in substantial spatial overlap with CG despite the observed environmental segregation at the extremities of their foraging trips (Bonnet-Lebrun in review).

## Conclusion

We found support for our hypothesis that BG abundance would decline with SST and further revealed that variation in SST was responsible for both their spatial distribution around the Iceland coast and their declining trends within sites. We rejected the hypothesis that changes in the numbers of CG competitors were driving a reduction in BG population size since both species experienced declines, albeit at different rates. However, this may simply mean that common environmental drivers are the dominating influence on the abundance of both species rather than that competition between them is absent. We found limited support for the hypothesis that BG partitioned their niches from CG, such that competition could arise when food becomes limiting owing to substantial overlap in space use and habitat. Such limitation has potential to arise where two species are declining to a lower equilibrium density owing to environmentally induced reductions in prey availability, as is the case for Iceland guillemots (Garðarsson, et al. 2019; Valdimarsson et al. 2012). We conclude that continued warming of the seas around Iceland is likely to cause continued declines of both guillemot species, and BG disproportionately so, but evidence for an additional role of competition is at present equivocal.

### Societal and policy implications

Seabirds play a significant role in Arctic food webs in terms of prey consumption (Barrett et al. 2006) and nutrient transport (Mosbech et al. 2018) as well as providing quarry for hunters (Merkel and Barry 2008) and natural spectacles that attract ecotourism (Burdon et al. 2017). Seabirds, therefore, deliver important ecosystem services to the Arctic, and their loss is of conservation and socioeconomic concern. Within an Icelandic context, continued warming will threaten guillemots and other seabird species with consequences for hunting and egging that are traditional aspects of Icelandic culture and identity. Reductions in the numbers of BG migrating to winter in Canada and Greenland may also increase the hunting pressures on resident populations of those countries with implications for sustainability (Frederiksen et al. 2019). Spectacular seabird colonies are among the natural attractions that have stimulated the rapid growth of ecotourism in Iceland, and declining populations will adversely affect income to remote coastal communities. Halting anthropogenic climate change will take a global effort and decades to achieve, but its effects on Icelandic seabirds may be partially offset by improved regulation of exploitation and pollution, which currently cause significant mortality in both the breeding and wintering areas (Frederiksen et al. 2019).

## Supplementary Information

Below is the link to the electronic supplementary material.

Supplementary file1 (PDF 1546 KB)
