# Supplementary material for: Cold comfort: Arctic seabirds find refugia from climate change and potential competition in marginal ice zones and fjords
Source: Ambio. 2021 Nov 9;51(2):345–54. doi: 10.1007/s13280-021-01650-7 (PMC8692633; doi:10.1007/s13280-021-01650-7)

Cold comfort: Arctic seabirds find refugia from climate change and potential competition in marginal ice zones and fjords: Supplementary Materials

Anne-Sophie Bonnet-Lebrun[1], Thomas Larsen[2], Thorkell Lindberg Thórarinsson[3], Yann Kolbeinsson[4], Morten Frederiksen[5], Tim I. Morley[1], Derren Fox[1], Aude Boutet[1], Fabrice le Bouard[1], Tanguy Deville[1], Erpur Snær Hansen[6], Thomas Hansen[7], Patrick Roberts[2] & Norman Ratcliffe[1]

[1]British Antarctic Survey (BAS), High Cross, Madingley Road, Cambridge, CB3 0ET, UK. email: notc@bas.ac.uk.

[2]Department of Archaeology, Max Planck Institute for the Science of Human History, Kahlaishce Str. 10, 07745 Jena, Germany. email: larsen@shh.mpg.de; roberts@shh.mpg.de.

[3]Icelandic Institute of Natural History, Garðabær, Iceland. email: thorkell.l.thorarinsson@ni.is.

[4]Northeast Iceland Nature Research Centre, Húsavík, Iceland. email: yann@nna.is.

[5]Department of Bioscience, Aarhus University, Denmark. email: mfr@bios.au.dk.

[6]South Iceland Nature Research Centre, Ægisgata 2. 900 Vestmannaeyjar, Iceland. Email: erpur@nattsud.is.

[7]GEOMAR Helmholtz-Zentrum für Ozeanforschung, ZLCA, Düsternbrooker Weg 20, 24105 Kiel. e-mail: thansen@geomar.de.

Supplement S1: Details of analytical methods

The log-transformed abundance of each guillemot species at a given time and location (n_{ti}) across all colonies in Iceland in 1983-1986 and 2005-2008 were taken from Garðarsson (1995) and Garðarsson et al. (2019). These were modelled in relation to cliff area (A_i; from Garðarsson 1995), competitor abundance (C_i: the numbers of the other species at the colony) and the average or minimum sea surface temperature (z_{ti}) within the maximum foraging area of each colony during each survey period.

The maximum foraging range from each colony was estimated from the total size of each colony during the 1983-1986 census (for both species combined) using equations in Gaston et al. (2013). A maps of foraging radii overlaid on SST rasters are presented in Supplementary Fig S2. The same radii were used for both survey periods, despite changes in population size, to prevent SST change being due to alteration in area sampled rather than a change with time. Daily Level 4 Operational SST and Sea Ice Analysis from the Group for High Resolution SST (resolution: 0.05˚x 0.05˚), provided by Copernicus Climate Change Service, were downloaded from https://cds.climate.copernicus.eu/ (doi.org/10.1038/s41597-019-0236-x). These were summarised from 1 May – 30 June during all years within each of the two census periods. This quantified the SST during each of the census periods and the change in SST that occurred between them, since the regime shift in Iceland occurred approximately halfway between (Valdimarsson *et al*. 2012). We used the average SST as a measure the prevailing environmental conditions across the foraging range, and the minimum SST to indicate the availability of colder patches of habitat within it. We fitted only one of these in any given model owing to their strong inter-correlation.

Within subject centring was used to disaggregate the effects of the time and space components of SST on abundance (Curran and Bauer 2011). The mean of values of SST across all time points within each colony ($\bar{z}_i$) were calculated and used as the variable to represent between-site variation. The within-site differences of SST at each time point from this site mean ($\dot{z}_{ti}$) were used to estimate within-site variation. A random intercept for site ($u0_i$) was fitted to accommodate repeated measures and remaining variance was the residual error (r_{ti}). Exploratory analysis did not find support for interactions among explanatory variables, so the global model containing all additive terms was:

$$n_{ti} = (\gamma 00 + \gamma 01\bar{z}_i + \gamma 10\dot{z}_{ti} + A_i + C_i) + (u0_i + r_{ti})$$

Adapted from Equation 12 in Curran and Bauer (2011) where γ_{00} is the coefficient for the intercept (or grand mean), γ_{01} is a direct estimate of the between-site effect, and γ_{10} is a direct estimate of the within-site effect. Linear mixed models (LMM), fitted in the R package nlme (Pinheiro *et al*. 2021), were used to estimate parameters and their uncertainty using an identity link and normal errors. We used normal errors rather than the Poisson errors typically used for counts as the data comprised large numbers with few zeros, and normal errors relaxed the assumption of variance equalling the mean. The proportion of BG within colonies was modelled in relation to minimum SST using the R package lmer (Bates *et al*. 2015) using a generalised LMM with a logit link and binomial errors to examine changes in relative abundance of the two species in relation to SST.

We classed sites according to the sector of Iceland in which they are situated (SW, NW, N, NE and SE). Papey and Skrúður in the SE were thus combined for further analysis. These are broad regional classifications that are also associated with different water masses and SST (see Study Sites section in main article). We modelled the response variables (trip distance, SST in foraging segments and isotope ratios) using general least squares (gls, where random effects were absent from models) or LMMs (where included; Zuur *et al.* 2009) with an identity link and normal errors, fitted using nlme. Explanatory fixed factors were species, colony and (for isotopes only) year, while random effects were individual (for trip distance and SST in foraging segments only). As there were missing site/species/year combinations for the stable isotope sampling, full factorial models could not be fitted, so each of the site-species-year combinations were expressed as levels of a single factor, which was fitted as a fixed effect in the model. As heteroscedasticity was evident among factor levels for all responses, we fitted identity variance structures to meet model assumptions and estimate differences in the variability among groups (the number of standard deviations relative to a reference level; SD_r) according to Zuur *et al*. (2009). In the case of SST in foraging segments, serial autocorrelation was evident in the residuals, so an order-one auto-regressive term was fitted within individual (Zuur *et al*. 2009).

In all analyses, Akaike's Information Criterion, adjusted for small sample size (AIC_c, using the R package AICcmodavg; Mazerolle 2020), was used for model selection and diagnostic plots (of normality, kurtosis, outliers, homoscedasticity and autocorrelation) and R^2 were used to confirm model

goodness of fit. For models with random effects, both marginal R^2_m (fixed effects alone) and the conditional R^2_c (fixed and random effects combined; Nakagawa and Schielzeth 2013) were calculated using the R package MuMln (Bartoń 2009). Tukey HSD was used to test differences between factor levels of interest (species, year and sites while controlling for each of the others) using the R package multComp (Hothorn *et al*. 2008).

References

Barton, K. 2009. Mu-MIn: Multi-model inference. R Package Version 0.12.2/r18. http://R-Forge.R-project.org/projects/mumin/

Bates D., M. Mächler, B. Bolker and S. Walker 2015. Fitting linear mixed-effects models using lme4. *Journal of Statistical Software* 67: 1–48. https://doi.org /10.18637/jss.v067.i01

Gaston, A.J., K.H. Elliott, Y. Ropert-Coudert, A. Kato, C.A. Macdonald, M.L. Mallory and H.G. Gilchrist 2013. Modeling foraging range for breeding colonies of thick-billed murres Uria lomvia in the Eastern Canadian Arctic and potential overlap with industrial development. *Biological Conservation* 168: 134-143. doi.org/10.1016/j.biocon.2013.09.018

Hothorn T., F. Bretz, and P. Westfall 2008. Simultaneous inference in general parametric models. *Biometrical Journal* 50: 346–363. https://doi.org/10.1002/bimj.200810425

Mazerolle, M.J. 2020. AICcmodavg: Model selection and multimodel inference based on (Q)AIC(c). R package version 2.3-1, https://cran.r-project.org/package=AICcmodavg

Nakagawa, S. and H. Schielzeth 2013. A general and simple method for obtaining R2 from generalized linear mixed-effects models. *Methods Ecology and Evolution* 4: 133-142. https://doi.org /10.1111/j.2041-210x.2012.00261.x

Pinheiro, J., D. Bates, S. DebRoy, D. Sarkar and R Core Team 2021. nlme: Linear and nonlinear mixed effects models. R package version 3.1-153, https://CRAN.R-project.org/package=nlme

Valdimarsson, H., O.S. Astthorsson, and J. Palsson 2012. Hydrographic variability in Icelandic waters during recent decades and related changes in distribution of some fish species. *ICES Journal of Marine Science* 69: 816-825. https://doi.org/10.1093/icesjms/fss027

Zuur, A., E.N. Ieno, N. Walker, A.A. Saveliev and G.M. Smith 2009. *Mixed effects models and extensions in ecology with R*. Springer. https://doi.org/10.18637/jss.v032.b01

Table S1: AIC_c values for spatiotemporal abundance of CG at colonies across the whole of Iceland during two surveys *c*. 21 years apart. The selected model is shown in bold. All models contain a random intercept of colony. For model names, A is area of the cliff, C the number of BG competitors. ta and tm are the average and minimum sea surface temperature within the foraging range of the colony during May and June, respectively, while the suffix w is time within sites and b between sites.

Model	K	AICc	Δ AICc	AICcWt	Cum.Wt	LL
taw+tab+A	6	68.38	0.00	0.38	0.38	-26.51
**taw+A**	**5**	**69.28**	**0.91**	**0.24**	**0.63**	**-28.49**
taw+tab+A+C	7	71.41	3.04	0.08	0.71	-26.37
taw+A+C	6	71.43	3.05	0.08	0.80	-28.03
tmw+A	5	71.80	3.43	0.07	0.87	-29.75
tmw+tmb+A	6	72.11	3.74	0.06	0.93	-28.38
tmw+A+C	6	72.72	4.34	0.04	0.97	-28.68
tmw+tmb+A+C	7	73.83	5.45	0.03	0.99	-27.58
tab+A	5	80.26	11.88	0.00	1.00	-33.97
tmw+tmb+C	6	80.59	12.21	0.00	1.00	-32.62
tmw+tmb	5	81.04	12.67	0.00	1.00	-34.37
tab+C+A	6	81.16	12.79	0.00	1.00	-32.90
A	4	81.39	13.01	0.00	1.00	-35.95
tmb+a	5	81.47	13.10	0.00	1.00	-34.58
taw	4	82.96	14.58	0.00	1.00	-36.74
A+C	5	83.14	14.77	0.00	1.00	-35.42
taw+tab	5	83.65	15.28	0.00	1.00	-35.67
tmw+C	5	83.80	15.42	0.00	1.00	-35.75
taw+C	5	83.95	15.57	0.00	1.00	-35.82
tmb+A+C	6	83.97	15.59	0.00	1.00	-34.31
taw+tab+C	6	84.96	16.58	0.00	1.00	-34.80
tmw	4	85.47	17.10	0.00	1.00	-38.00
tmb	4	90.63	22.25	0.00	1.00	-40.57
tmb+C	5	90.78	22.40	0.00	1.00	-39.24
C	4	93.81	25.44	0.00	1.00	-42.17
tab+C	5	94.74	26.37	0.00	1.00	-41.22
Null	3	95.26	26.89	0.00	1.00	-44.20
tab	4	95.76	27.38	0.00	1.00	-43.14

Table S2: AIC_c values for spatiotemporal abundance of BG at colonies across the whole of Iceland during two surveys *c*. 21 years apart. See Table S1 for model codes.

Model	K	AICc	Δ AICc	AICcWt	Cum.Wt	LL
**tmw+tmb**	**5**	**134.43**	**0.00**	**0.46**	**0.46**	**-61.06**
tmw+tmb+C	6	135.85	1.42	0.23	0.69	-60.24
tmw+tmb+A	6	136.36	1.93	0.18	0.86	-60.50
tmw+tmb+A+C	7	139.13	4.70	0.04	0.91	-60.23
taw+tab+A	6	140.01	5.58	0.03	0.94	-62.33
tmb	4	140.99	6.56	0.02	0.95	-65.76
tmb+C	5	141.40	6.97	0.01	0.97	-64.55
taw+tab+C	6	142.08	7.65	0.01	0.98	-63.36
tmb+a	5	142.70	8.27	0.01	0.98	-65.20
taw+tab+A+C	7	143.06	8.63	0.01	0.99	-62.20
tmb+A+C	6	144.36	9.93	0.00	0.99	-64.50
taw+tab	5	144.87	10.44	0.00	1.00	-66.28
tab+C	5	146.44	12.01	0.00	1.00	-67.06
taw+A	5	147.47	13.04	0.00	1.00	-67.58
taw+C	5	148.22	13.79	0.00	1.00	-67.96
tab+A	5	148.34	13.91	0.00	1.00	-68.02
tmw+C	5	148.52	14.09	0.00	1.00	-68.11
tab+C+A	6	149.21	14.78	0.00	1.00	-66.93
tmw+A	5	149.46	15.03	0.00	1.00	-68.58
taw+A+C	6	149.86	15.43	0.00	1.00	-67.25
taw	4	150.18	15.75	0.00	1.00	-70.35
tmw+A+C	6	150.84	16.41	0.00	1.00	-67.74
tmw	4	152.17	17.74	0.00	1.00	-71.35
C	4	152.21	17.78	0.00	1.00	-71.37
tab	4	153.42	18.99	0.00	1.00	-71.97
A+C	5	155.03	20.60	0.00	1.00	-71.36
A	4	156.02	21.59	0.00	1.00	-73.27
Null	3	158.93	24.50	0.00	1.00	-76.04

Table S3: AICc tables for the responses of foraging range, SST in foraging segments (from bird-borne loggers) and isotope ratios of carbon and nitrogen. In the case of isotope ratios each-species-site-year combination is expressed as level in a single factor so effects cannot be disaggregated by component variables. The differences among these levels according to Tukey HSD tests are shown in Table S5. Selected models are shown in bold text.

Response	Mean	SD	K	AICc	ΔAICc	AICcWt	Cum.Wt	LL
	**Site**	**Site**	**9**	**2158.26**	**0.00**	**0.35**	**0.35**	-1069.94
	Site*Sp	Site	13	2158.29	0.02	0.35	0.70	-1065.76
	Site+Sp	Site	10	2158.58	0.32	0.30	1.00	-1069.06
Foraging	Species	Site	7	2219.25	60.99	0.00	1.00	-1102.51
Range	Site	Null	6	2254.23	95.97	0.00	1.00	-1121.03
	Site+Sp	Null	7	2256.20	97.94	0.00	1.00	-1120.99
	Species	Null	10	2256.31	98.05	0.00	1.00	-1117.92
	Null	Null	3	2310.99	152.72	0.00	1.00	-1152.47
	Species	Null	4	2312.36	154.09	0.00	1.00	-1152.14
	**Site*Sp**	**Site**	**14**	**3623.69**	**0.00**	**1.00**	**1.00**	**-1797.70**
	Site+Sp	**Site**	11	3640.16	16.47	0.00	1.00	-1808.99
Foraging	Site	Site	10	3654.48	30.79	0.00	1.00	-1817.17
SST	Sp	Site	8	3829.68	205.99	0.00	1.00	-1906.79
	Site*Sp	Null	11	4886.77	1263.08	0.00	1.00	-2432.29
	Site	Null	7	4889.91	1266.22	0.00	1.00	-2437.92
	Site+Sp	Null	8	4891.80	1268.11	0.00	1.00	-2437.85
	Sp	Null	5	5017.19	1393.50	0.00	1.00	-2503.57
	Null	Null	4	5022.38	1398.69	0.00	1.00	-2507.18
	**Site*Sp*Yr**	**Site*Sp*Yr**	**32**	**277.96**	**0.00**	**1.00**	**1.00**	**-101.73**
$\delta^{13}C$	Site*Sp*Yr	Null	17	384.81	106.85	0.00	1.00	-173.99
	Null	Site*Sp*Yr	17	393.51	115.55	0.00	1.00	-178.34
	Null	Null	2	494.49	216.52	0.00	1.00	-245.22
	**Site*Sp*Yr**	**Site*Sp*Yr**	**32**	**277.96**	**0.00**	**1.00**	**1.00**	**-101.73**
$\delta^{15}N$	Site*Sp*Yr	Null	17	384.81	106.85	0.00	1.00	-173.99
	Null	Site*Sp*Yr	17	393.51	115.55	0.00	1.00	-178.34
	Null	Null	2	494.49	216.52	0.00	1.00	-245.22

Table S4. Estimates of $\delta^{13}C$ and $\delta^{15}N$ in guillemot blood cells from the minimum adequate linear mixed models with one SE and estimates of relative variability (SD_r; scaled to common guillemots in the NE in 2018). N is sample size and the Z scores and P values show significance of Tukey HSD adjusted differences between species within years and sites. Sample sizes in 2019 in the NE were inadequate for statistical testing, while no BG were sampled in the SW or SE in 2018 and no samples from either species were collected in the SW in 2019.

**Isotope**	**Sector**	**Species**	**Year**	**N**	**Mean**	**SE**	**SD_r**	**z**	**P**
$\delta^{13}C$	SW	CG	2018	7	-19.3	0.06	0.80	-	-
$\delta^{13}C$	NW	BG	2018	15	-20.1	0.11	2.26	2.19	< 0.005
$\delta^{13}C$	NW	CG	2018	15	-20.5	0.07	1.42		
$\delta^{13}C$	N	BG	2018	15	-20.1	0.04	0.89	7.95	< 0.001
$\delta^{13}C$	N	CG	2018	15	-20.3	0.04	0.78		
$\delta^{13}C$	NE	BG	2018	15	-20.0	0.03	0.65	0.13	> 0.8
$\delta^{13}C$	NE	CG	2018	15	-20.0	0.05	1.00		
$\delta^{13}C$	SE	CG	2018	15	-20.1	0.11	1.32	-	-
$\delta^{13}C$	N	BG	2019	9	-19.7	0.04	0.62	1.32	> 0.1
$\delta^{13}C$	N	CG	2019	15	-19.7	0.03	0.62		
$\delta^{13}C$	NE	BG	2019	3	-21.5	0.08	0.72	-	-
$\delta^{13}C$	NE	CG	2019	2	-21.3	0.03	0.22		
$\delta^{13}C$	NW	BG	2019	31	-19.8	0.10	2.87	6.45	< 0.0001
$\delta^{13}C$	NW	CG	2019	32	-20.6	0.08	2.34		
$\delta^{13}C$	SE	BG	2019	10	-19.5	0.15	2.40	2.98	< 0.005
$\delta^{13}C$	SE	CG	2019	14	-20.1	0.11	2.20		
$\delta^{15}N$	SW	CG	2018	7	13.2	0.13	1.54	-	-
$\delta^{15}N$	NW	BG	2018	15	12.6	0.17	2.85	3.84	< 0.0005
$\delta^{15}N$	NW	CG	2018	15	11.8	0.11	1.95		
$\delta^{15}N$	N	BG	2018	15	11.8	0.04	0.74	0.38	> 0.7
$\delta^{15}N$	N	CG	2018	15	11.8	0.05	0.88		
$\delta^{15}N$	NE	BG	2018	15	12.1	0.05	0.80	0.83	> 0.4
$\delta^{15}N$	NE	CG	2018	15	12.2	0.06	1.00		
$\delta^{15}N$	SE	CG	2018	15	13.1	0.20	3.39	-	-
$\delta^{15}N$	N	BG	2019	9	11.6	0.06	0.82	0.56	> 0.5
$\delta^{15}N$	N	CG	2019	15	11.5	0.07	1.34		
$\delta^{15}N$	NE	BG	2019	3	11.5	0.02	0.18	-	-
$\delta^{15}N$	NE	CG	2019	2	11.9	0.02	0.11		
$\delta^{15}N$	NW	BG	2019	31	12.2	0.10	2.85	3.71	< 0.0005
$\delta^{15}N$	NW	CG	2019	33	11.6	0.12	3.04		
$\delta^{15}N$	SE	BG	2019	10	11.8	0.18	2.94	1.99	< 0.05
$\delta^{15}N$	SE	CG	2019	15	12.4	0.25	4.33		

Table S5. Tukey HSD multiple pairwise comparison tests of differences in mean $\delta^{13}C$ and $\delta^{15}N$ in guillemot blood cells among geographic sectors of Iceland within species and year.

				**$\delta^{13}C$**		**$\delta^{15}N$**	
**Species**	**Year**	**Sector1**	**Sector2**	**z**	**P**	**z**	**P**
CG	2018	SW	NW	13.12	< 0.0001	7.92	< 0.0001
CG	2018	SW	N	15.27	< 0.0001	10.31	< 0.0001
CG	2018	SW	NE	9.92	< 0.0001	7.21	< 0.0001
CG	2018	SW	SE	0.80	> 0.4	0.43	> 0.6
CG	2018	NW	N	1.66	> 0.05	4.8	< 0.0001
CG	2018	NW	NE	5.10	< 0.0001	2.66	< 0.01
CG	2018	NW	SE	13.14	< 0.0001	5.59	< 0.0001
CG	2018	N	NE	4.86	< 0.0001	5.23	< 0.0001
CG	2018	N	SE	14.87	< 0.0001	6.63	< 0.0001
CG	2018	NE	SE	10.04	< 0.0001	4.54	< 0.0001
CG	2019	NW	N	10.67	< 0.0001	5.33	< 0.0001
CG	2019	NW	NE	7.66	< 0.0001	1.80	> 0.05
CG	2019	NW	SE	4.17	< 0.0001	2.88	< 0.005
CG	2019	N	NE	38.47	< 0.0001	4.45	< 0.0001
CG	2019	N	SE	2.76	< 0.001	6.63	< 0.0001
CG	2019	NE	SE	10.49	< 0.0001	4.54	< 0.0001
BG	2018	NW	N	0.58	> 0.5	4.81	< 0.0001
BG	2018	NW	NE	0.38	> 0.7	2.86	< 0.005
BG	2018	N	NE	2.10	< 0.05	5.23	< 0.0001
BG	2019	NW	N	1.33	> 0.1	4.81	< 0.0001
BG	2019	NW	NE	21.04	< 0.0001	2.86	< 0.005
BG	2019	NW	SE	1.74	< 0.0001	1.91	> 0.05
BG	2019	N	NE	21.04	< 0.0001	1.53	> 0.1
BG	2019	N	SE	1.10	> 0.2	1.25	> 0.2
BG	2019	NE	SE	12.26	< 0.0001	1.87	> 0.05

Table S6. Tukey HSD multiple pairwise comparison tests of differences in mean $\delta^{13}C$ and $\delta^{15}N$ in guillemot blood cells between 2018 and 2019 within species and geographic sectors of Iceland.

		$\delta^{13}C$		δN^{15}	
Species	Sector	z	P	z	P
CG	NW	1.64	> 0.1	1.17	> 0.2
CG	N	12.49	< 0.0001	2.48	< 0.05
CG	NE	21.86	< 0.0001	5.16	< 0.0001
CG	SE	6.60	< 0.0001	2.07	< 0.05
BG	NW	1.73	> 0.05	2.01	< 0.05
BG	N	7.96	< 0.0001	2.48	< 0.05
BG	NE	17.62	< 0.0001	11.86	< 0.0001

Figure S1. Summary of timing and duration of individual tracks by species, site and stage of breeding season. Bars represent the period for which each individual was tracked and the numbers by the bars are the number of trips the bird made during the deployment.

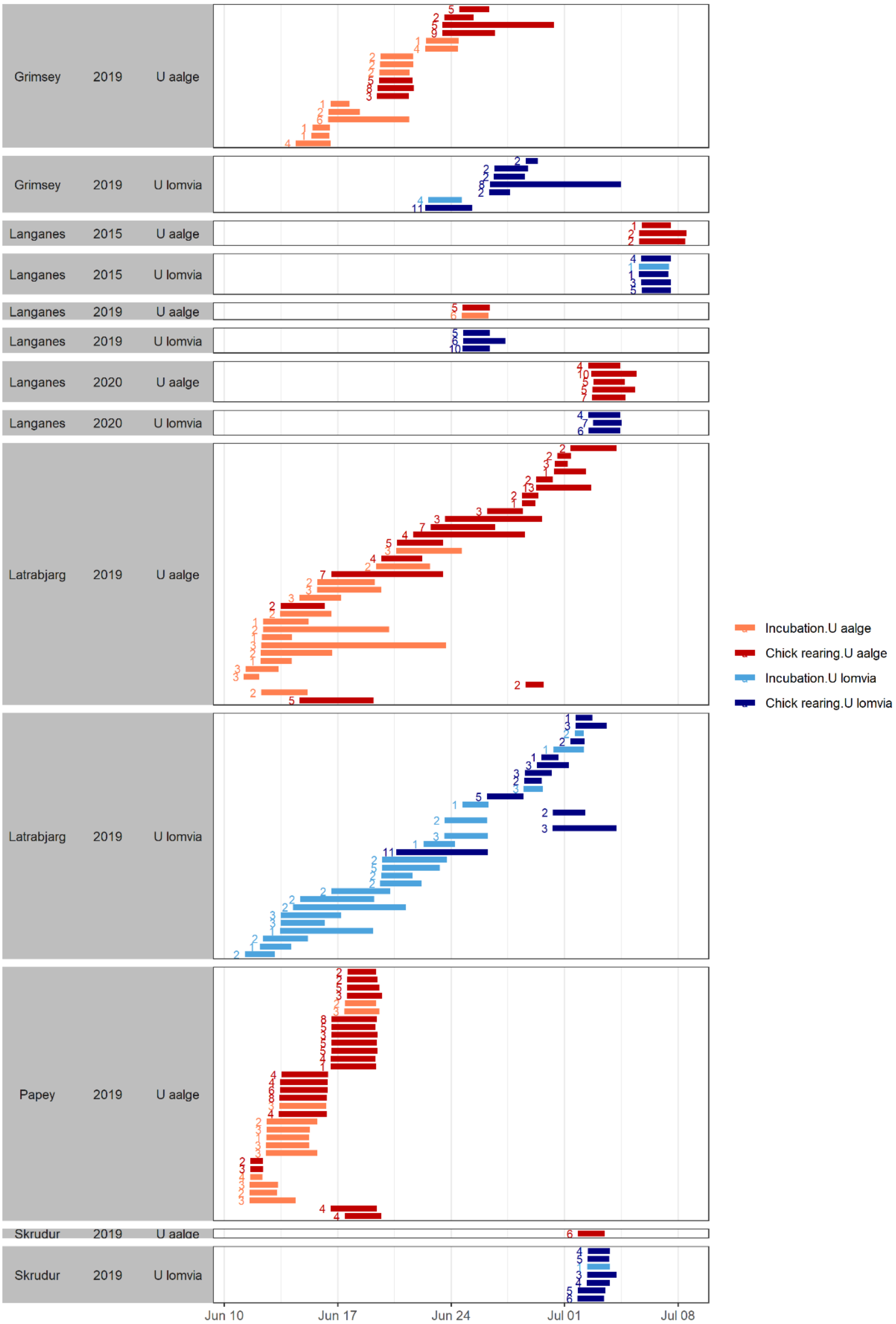

Fig S2: Derivation of SSTs for colonies according to their location, size (numbers of both guillemot species combined) and survey period for use in the models of abundance. Top figures show the relative number of birds (pie chart size) and proportion of BG (lighter segments) for all colonies in Iceland during the two census periods. Lower figures show inferred maximum foraging range from each colony estimated from population size. SST data were taken from the Copernicus Climate Change Service (see Supplement S1 for full details).

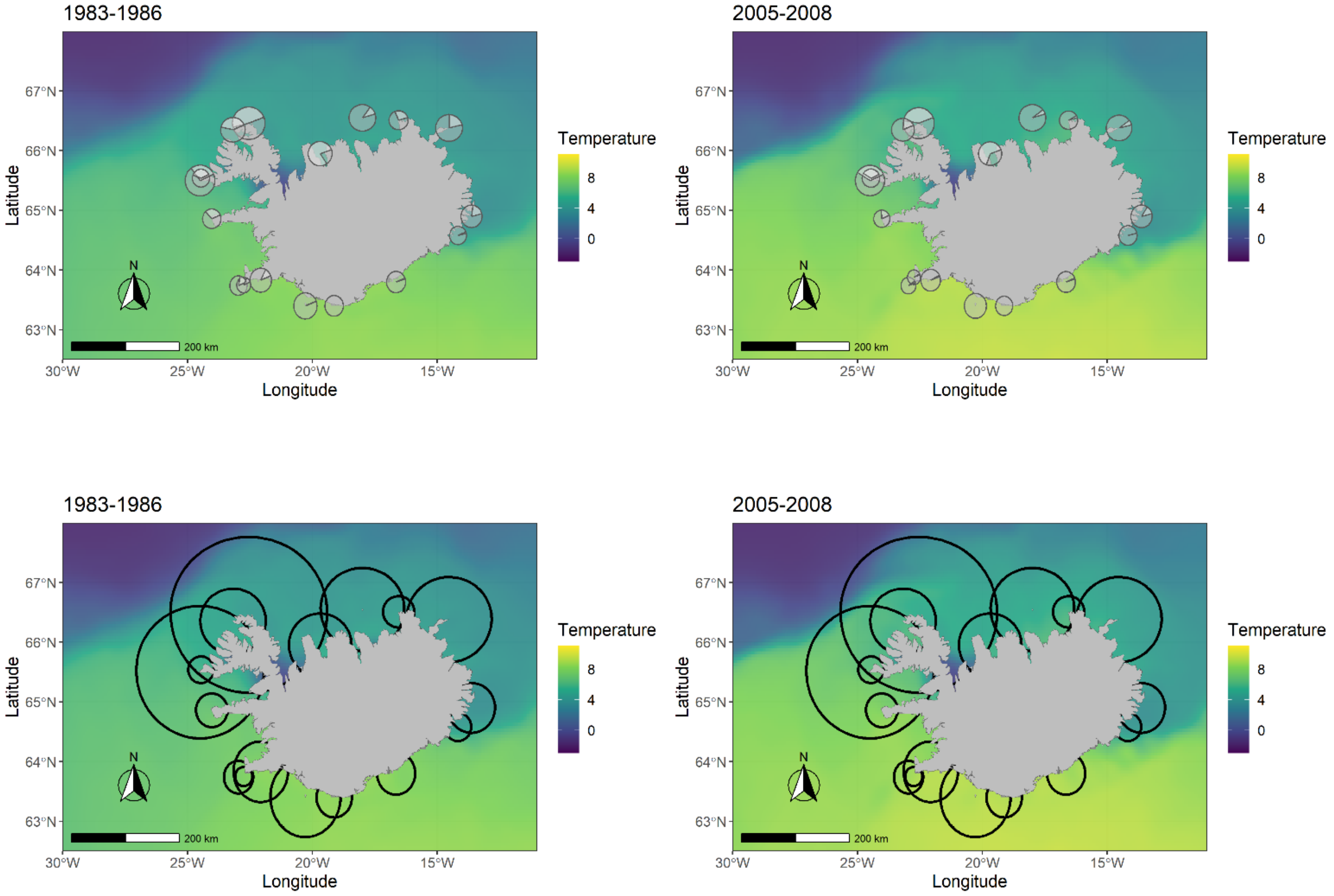

Fig. S3: Details of tracks at individual colonies. A. Grimsey (left) and Langanes; B. Latrabjarg, C. Papey (lower) and Skrúður.

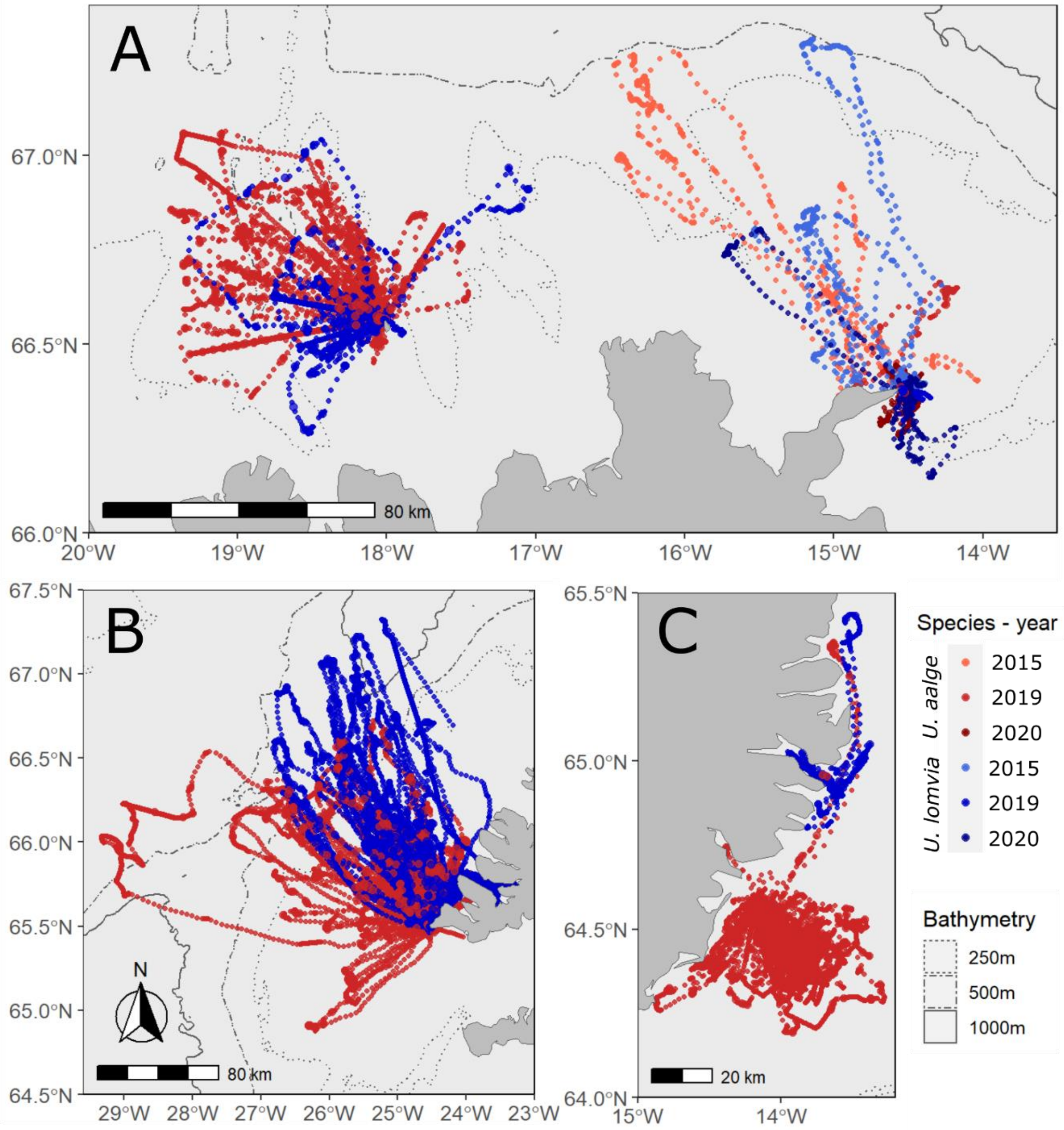

Fig. S4. Split Violin plots showing differences in isotope ratios between years within species and sites

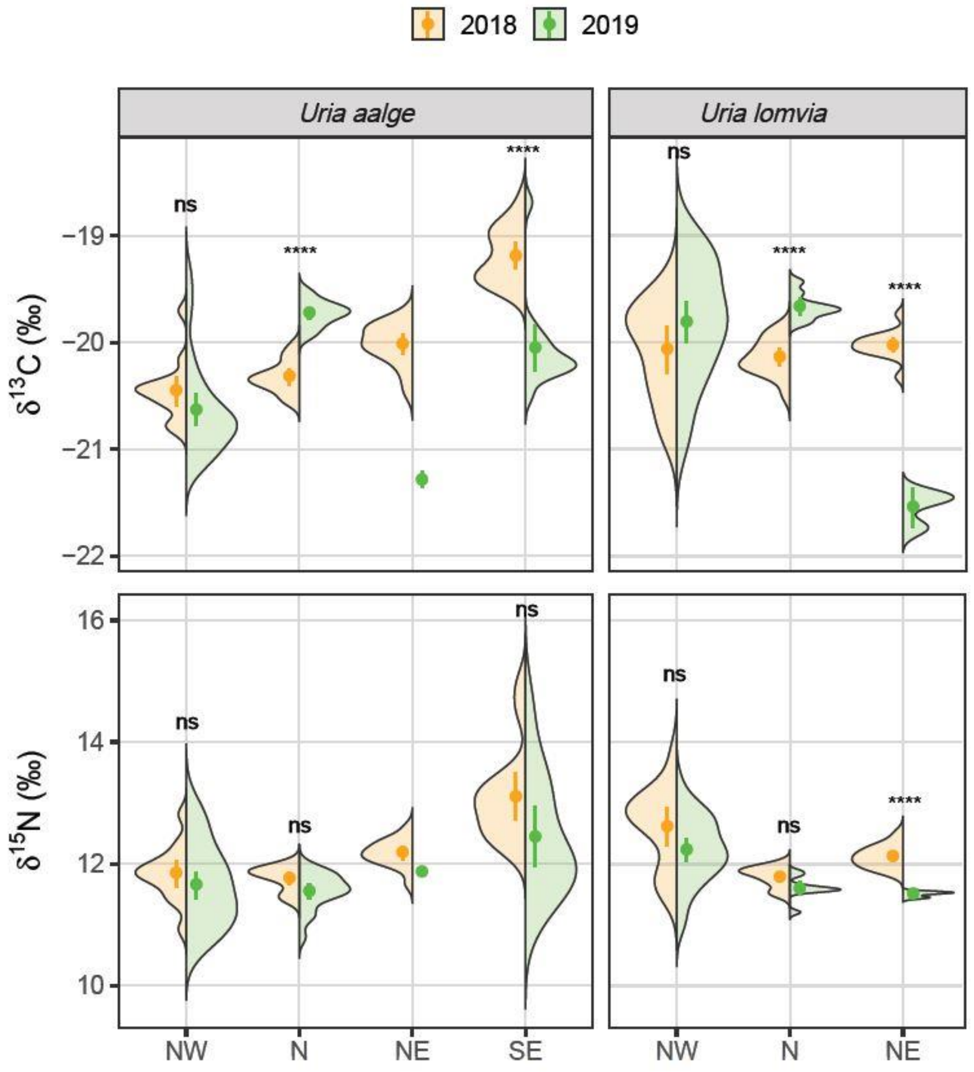

Fig. S5. Changes in SST around the coast of Iceland in 2019 compared to 2018.

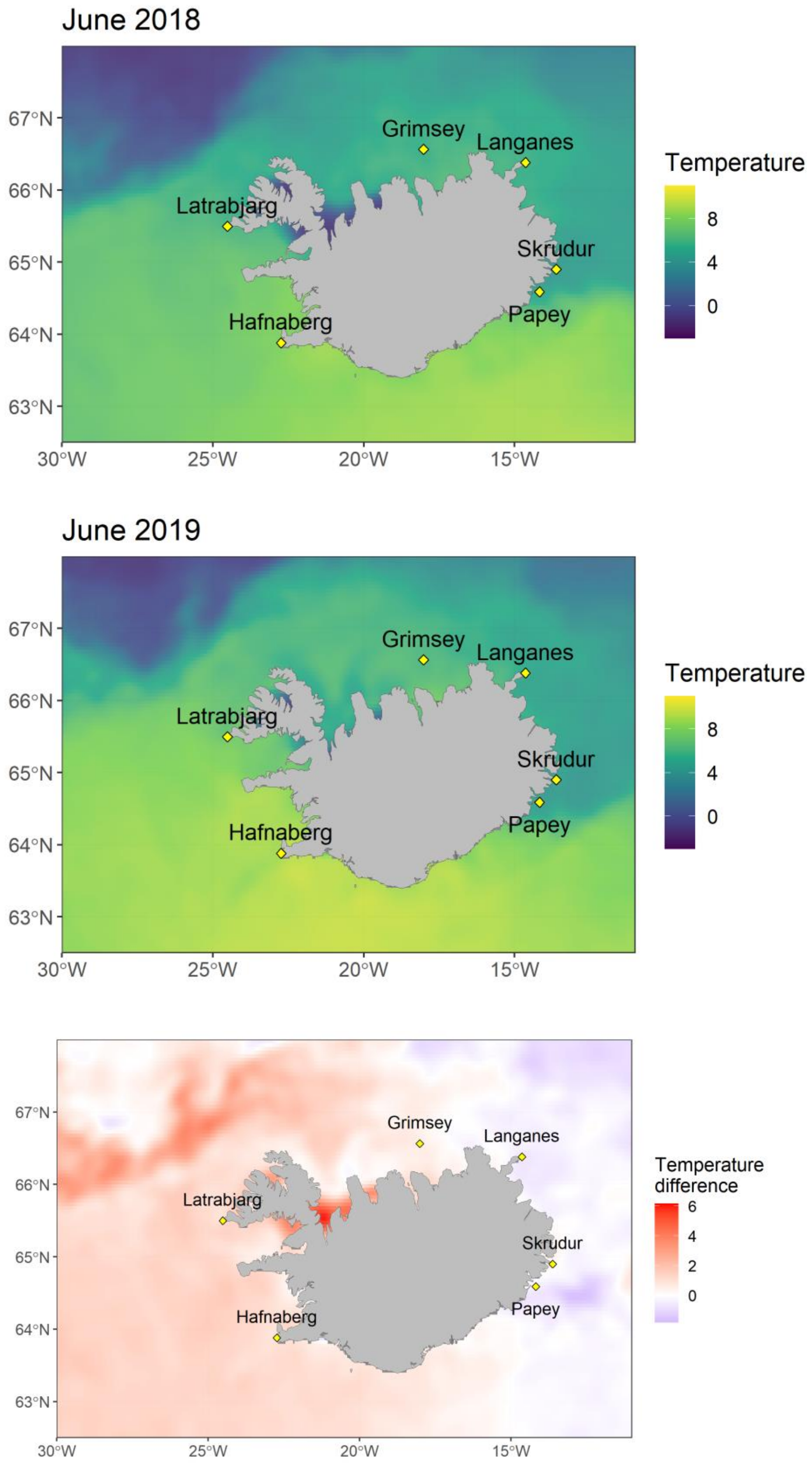

Supplement: Supplementary file 1 — Supplementary file1 (PDF 1546 KB) [file 13280_2021_1650_MOESM1_ESM.pdf]
